# Clinical efficacy and prognostic factors of regenerative endodontic procedure for immature permanent teeth with periapical periodontitis

**DOI:** 10.3389/fmed.2026.1945140

**Published:** 2026-10-05

**Authors:** Huan Jiang, Siyu Bai, Ping Chen

**Affiliations:** 1 Department of Pediatric Dentistry, Sichuan Hospital of Stomatology, Chengdu, Sichuan, China; 2 Department of Dental, Sichuan Jinxin Xinan Women & Children Hospital, Chengdu, Sichuan, China; 3 Department of Stomatology, The First Affiliated Hospital of Changsha Medical University, Changsha, Hunan, China

**Keywords:** clinical efficacy, immature permanent teeth, influence factor, periapical periodontitis, regenerative endodontic procedure

## Abstract

**Objective:**

To investigate the clinical effects of regenerative endodontic procedures in the treatment of immature permanent teeth with periapical periodontitis and to analyze the factors influencing prognosis.

**Methods:**

Clinical data of 148 immature permanent teeth with periapical periodontitis treated at our hospital from January 2022 to December 2025 were retrospectively analyzed. The patients were equally divided into Group A (regenerative endodontic procedure, *n* = 74) and Group B (apexification, *n* = 74). Clinical efficacy, root development, periodontal parameters, gingival crevicular fluid VEGF levels, and complication rates were compared. All subjects underwent 6-month follow-up and were stratified into good and poor prognosis subgroups. Multivariate logistic regression was used to screen for prognostic factors.

**Results:**

After 6 months of therapy, Group A achieved a superior overall response rate, thicker root canal walls, longer root lengths, lower SBI, PLI, and PD, elevated gingival crevicular fluid bFGF and VEGF levels, and fewer complications (*P* < 0.05). All participants completed the 6-month follow-up and were categorized into poor-prognosis (*n* = 26) and good-prognosis (*n* = 122) cohorts. The poor-prognosis group had fewer regenerative endodontic procedure cases and larger apical lesion areas (*P* < 0.05). Multivariate logistic regression identified treatment modality and apical lesion size as independent risk factors for an unfavorable prognosis (*P* < 0.05).

**Conclusion:**

Regenerative endodontic procedures are associated with favorable short-term (6-month) outcomes in immature permanent teeth with apical periodontitis, including improved root development and repair, a more favorable periodontal microenvironment, increased levels of repair-related cytokines, and fewer complications. The treatment modality and extent of the apical lesion were associated with prognosis. Individualized treatment decisions should consider the apical lesion characteristics and clinical status of the affected tooth to optimize outcomes in pediatric patients.

Immature permanent teeth are characterized by undeveloped roots, open apical foramen, and thin root canal walls. They are special and important dental tissues in the mouth of children and adolescents and play a key role in maxillofacial development, occlusal establishment, and maintenance of complete dentition (1). Trauma, deep caries infection, and abnormal tooth development are the main causes of pulp necrosis and periapical periodontitis in immature permanent teeth. Poor oral hygiene aggravates theprogression of caries and increases the risk of disease. Delayed treatment hinders root development and causes root deformity, recurrent swelling and pain, jaw lesions, dentition imbalance, and other problems. Impaired root maturation may lead to abnormal tooth eruption and occlusal disharmony, and recurrent periapical infection contributes to masticatory dysfunction, chronic oral pain, and poor quality of life, which seriously endangers the oral health, maxillofacial development, and physical and mental health of teenagers (2, 3). Periapical periodontitis of immature permanent teeth is more difficult to treat than that of mature permanent teeth, and the prognosis is difficult to control. Traditional therapies have several limitations. Therefore, exploring minimally invasive and efficient technology that can control infection, promote root development, and facilitate tooth regeneration is the research and clinical focus in the field of dental endodontics in children (4). Apexification can clear root canal infection, control apical inflammation, relieve pain, induce the formation of a calcification barrier in the apical region, and slightly increase the length of the root. However, this treatment cannot reconstruct the pulp activity and root canal blood supply, cannot form a physiological apical structure, and is difficult to thicken the root canal wall, which can easily cause a decline in tooth fracture resistance and increase the risk of tooth fracture, and the prognosis is poor (5). Endodontic revascularization relies on the regenerative ability of dental papilla stem cells and periodontal ligament stem cells that survive in the apical region. Through standardized treatment, root canal blood supply can be reconstructed and tissue regeneration induced, which can promote root development and apical foramen closure, retain tooth development potential, and simultaneously realize infection control, tissue repair, and functional regeneration (6). Most existing studies focus on the comparison of the curative effect of revascularization and traditional surgery or the influence of a single factor on the therapeutic effect, and lack an analysis of the relevant factors affecting prognosis. According to the American Association of Endodontists (AAE) Glossary of Endodontic Terms (10th edition,2020), the historical term “revascularization” is currently termed regenerative endodontic procedure, which refers to biologically-based procedures designed to physiologically replace damaged dentin-pulp complex structures (7). Based on the above clinical status and research gap, this study aimed to explore the clinical efficacy and prognostic factors of immature permanent teeth with periapical periodontitis revascularization and provide a reference for the standardized diagnosis and treatment of similar diseases.

## 1 Data and methods

### 1.1 General information

This study was approved by the Ethics Committee of the Sichuan Hospital of Stomatology (Ethics approval No.: 2026010008). Clinical data from 148 immature permanent teeth in 148 children with apical periodontitis who were treated at our hospital between January 2022 and December 2025 were retrospectively collected. Baseline characteristics were compared between the groups to assess their comparability. However, baseline comparisons alone cannot eliminate confounding or selection bias, and residual confounding remains an inherent limitation of this study. The patients were divided into group A (regenerative endodontic procedure, *n* = 74) and group B (apexification, *n* = 74), with one affected tooth included in each patient. Treatment allocation was non-randomized and determined by the attending pediatric endodontists based on a comprehensive clinical and cone-beam computed tomography (CBCT) assessment, including apical foramen dimensions, periapical lesion size, patient age, duration of pulp necrosis, and the anticipated risks and benefits of regenerative endodontic treatment. Teeth considered to have a favorable apical stem-cell microenvironment were treated with regenerative endodontic procedures, whereas apexification was selected for cases considered to be at higher risk of regenerative treatment failure. Given the retrospective, nonrandomized treatment allocation, treatment selection bias cannot be excluded.

Group B comprised 43 males and 31 females, with an average age of (11.70 ± 2.32) years (range, 6–15 years). The course of the disease was 4–9 days, with an average of (6.53 ± 1.11) days. Group A comprised 40 males and 34 females, aged 5–15 years, with an average age of (12.01 ± 2.40) years. The course of disease was 3–9 days, with an average of (6.38 ± 1.26) days. There were no significant differences in the clinical data between the two groups. Inclusion criteria were as follows: ① meet the diagnostic criteria of pediatric dentistry (8): teeth with repeated swelling and pain, occlusal discomfort, and gum abscess; oral imaging examination showed periapical bone transmission shadow, incomplete root development, and open apical foramen; ② the teeth were immature permanent teeth with incomplete root development and apical foramen unclosed after eruption; ③ no severe tooth defect: tooth structure loss < 1/2 clinical crown, which was in line with the indications of conservative treatment and could be restored after endodontic therapy; ④ the affected tooth received endodontic treatment for the first time, with no history of root canal retreatment or apical surgery on this tooth; and ⑤ the clinical data were complete and the follow-up could be completed. The exclusion criteria were as follows: ① complicated with coagulation dysfunction; ② longitudinal root fractures and large tooth defects (tooth structure loss ≥1/2 clinical crown) in the affected teeth, which could not be retained; ③ acute suppurative periapical periodontitis with maxillofacial space infection and systemic fever; ④ severe periodontal disease defined as probing depth ≥6 mm combined with clinical attachment loss ≥3 mm; other pulp diseases; ⑤ low immune function: patients with confirmed immunodeficiency diseases or long-term immunosuppressant medication; and ⑥ inability to tolerate oral surgery.

A total of 217 medical records of children with immature permanent teeth and periapical periodontitis treated between January 2022 and December 2025 were screened for eligibility. Of these, 69 records were excluded for the following reasons: multiple eligible teeth from the same patient (*n* = 8), incomplete clinical data or unavailable 6-month follow-up records (*n* = 22), and fulfillment of the predefined exclusion criteria, including longitudinal root fracture, extensive tooth defects that precluded treatment, systemic immunodeficiency, maxillofacial space infection, or inability to tolerate endodontic procedures (*n* = 39). Finally, 148 teeth from 148 unique patients were included in this retrospective study, with one tooth per patient. The patients were divided into Group A (regenerative endodontic procedure, *n* = 74) and Group B (apexification, *n* = 74). The study selection process is illustrated in Figure 1.

**Figure 1:**
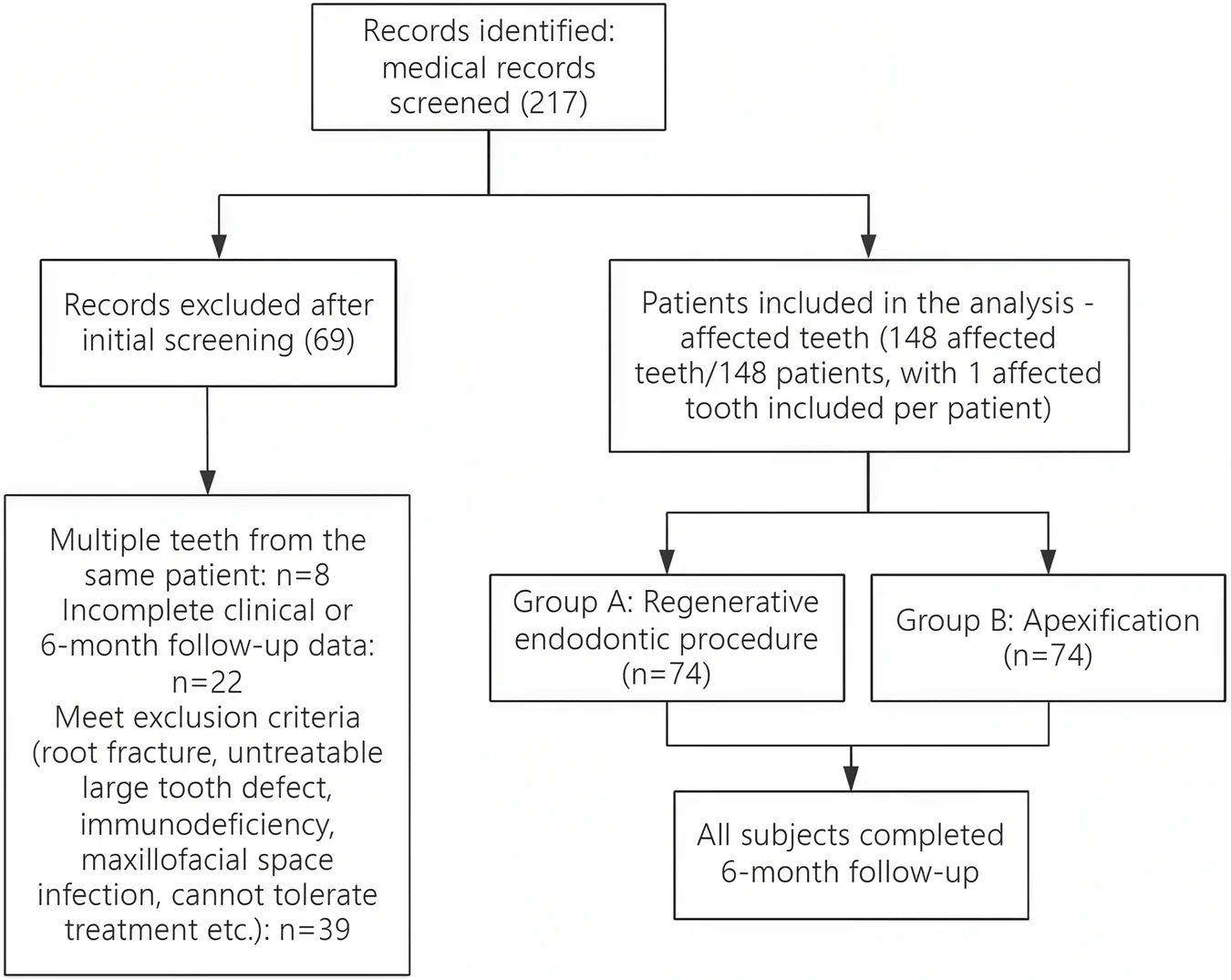
Case screening process diagram.

### 1.2 Method

All procedures included in the study were performed by senior attending physicians or physicians of higher rank with > 5 years of clinical experience in the Department of Pediatric Stomatology at our hospital. All operators received standardized training in regenerative endodontic treatment and followed uniform treatment protocols and procedural checklists to minimize operator-related variations. Cone-beam computed tomography (CBCT) was performed in all patients before the treatment.The open diameter of the apical foramen, area of periapical bone destruction, initial root length, and thickness of the root canal wall were measured, and the development status of the root, range of inflammatory invasion, and value of tooth retention were evaluated. An individualized treatment plan was formulated according to the children's age, oral health status, and trauma history. After treatment, all children were given standardized anti-infection, oral health guidance, and a standardized follow-up plan to ensure the balance and reliability of the two groups of research data to the greatest extent.

Group B underwent conventional apexification as follows: 1) After routine oral disinfection with iodophor and local infiltration anesthesia, an access cavity was prepared using a high-speed handpiece and round bur. The roof of the pulp chamber was completely removed to expose the root canal orifice, and the necrotic pulp and inflammatory granulation tissues within the pulp chamber and root canal were thoroughly removed. (2) After cleaning and drying the root canal, freshly prepared calcium hydroxide paste was slowly introduced into the canal and placed to ensure adequate contact with the root canal walls and the apical region. (3) The access cavity was temporarily sealed using zinc oxide–eugenol cement. Patients were scheduled for follow-up visits every three months. At each visit, the temporary restoration was removed, the root canal was cleaned to remove the previous medicament and any exudate, and fresh calcium hydroxide paste was applied. ④ Periapical bone healing and apical barrier formation were radiographically evaluated at 3-month intervals. Complete apical barrier formation was radiographically defined as a continuous mineralized hard-tissue bridge across the apical foramen, stable apical morphology, and absence of progressive periapical radiolucency. Once apical barrier formation was confirmed radiographically, conventional root canal obturation and permanent resin restoration were performed on the tooth. The 6-month follow-up period was calculated from the date of definitive root canal obturation, rather than from the initial apexification procedure. Some patients required multiple calcium hydroxide replacements before completing apical barrier formation.

Group A was treated with regenerative endodontic procedure: ① The basic treatment process of preoperative disinfection, local anesthesia, pulpotomy and roof opening, root canal debridement, alternative irrigation, root canal drying was the same as that of group B, and the necrotic infected pulp tissue in the root canal was completely removed to eliminate the active inflammation in the root canal. ② The root canals were gently dredged with 10/15 small K files, and only slight root-canal patency treatment was performed without excessive mechanical preparation to maximize the protection of root-canal-wall dentin, dental papilla stem cells, and periapical granulation tissue. Irrigation protocol: 3 mL 3% hydrogen peroxide followed by 3 mL sterile normal saline for each cycle; 3-5 repeated cycles. Irrigation was performed under low pressure with a 27-gauge side-vented needle placed 2-3 mm short of the apical foramen. The total irrigation volume ranged from 18 to 30 mL. After rinsing, the root canals were dried using sterile paper tips. ③ Calcium hydroxide preparation was embedded in the root canal and evenly applied to the root canal wall to achieve continuous antibacterial disinfection in the root canal. Zinc oxide clove oil cement was used to tightly and temporarily seal the tooth cavity. The children were asked to return for a follow-up visit within 1–2 weeks. During the visit, discomfort symptoms such as pain, gingival redness and swelling, and sinus discharge were observed. ④ After the follow-up examination showed that the affected teeth had no symptoms, negative tapping pain, complete closure of the gingival sinus, and no inflammatory exudation, the temporary sealing materials and drugs in the root canal were removed, and the root canal was rinsed and dried alternately again to ensure a sterile environment in the root canal. ⑤ The aseptic root canal file was used to penetrate the apical foramen to 2–3 mm in the periapical tissue, induce periapical tissue bleeding, guide blood countercurrent to fill the middle and lower segments of the root canal, and allow it to stand for 10–15 min until a uniform and stable blood clot formed in the root canal, providing a scaffold basis for dental pulp regeneration. ⑥ After the blood clot is completely formed and stabilized, cover the root canal orifice with mineral trioxide condensate (Dentsply SIRONA; ProRoot MTA white) to a thickness of 2–3 mm. The material was mixed according to the standard proportion in the product manual and gently compacted and trimmed to ensure that the root canal orifice was tightly closed and isolated from oral bacteria. Glass ionomer cement was used as the bottom, and double-layer resin was filled and polished in layers to complete the permanent restoration of teeth and restore the occlusal morphology of the affected teeth.

The regenerative endodontic procedure generally followed the AAE 2025 Regenerative Endodontics Position Statement, including minimal canal instrumentation, induction of apical bleeding, formation of a blood clot scaffold, and bioceramic coronal sealing. The main deviation from the AAE recommendations was the use of 3% hydrogen peroxide combined with normal saline for root canal irrigation instead of a sodium hypochlorite-based irrigation.

In this retrospective clinical cohort study, irrigation with 3% hydrogen peroxide combined with normal saline under low pressure was used to minimize the potential risk of cytotoxic effects on periapical tissues and stem cells in immature teeth with open apices. Given the thin root canal walls and open apices of immature permanent teeth, this protocol was designed to reduce periapical tissue irritation. However, sodium hypochlorite is the irrigant recommended in the current AAE guidelines, and the use of an alternative irrigation protocol should be considered a limitation of the present study.

The clinical workflows differed between the two groups. Regenerative endodontic procedures are generally completed within two visits, whereas apexification requires repeated calcium hydroxide replacement at 3-month intervals until radiographic evidence of apical barrier formation is achieved. To standardize the outcome assessment period, a 6-month follow-up interval was calculated from the date of definitive coronal restoration or root canal obturation in both groups, rather than from the initial treatment visit. All patients were followed up for 6 months after treatment, and no cases were lost. All follow-ups werethe responsibility of a specially assigned person, and the unified follow-up standard shall be strictly followed. The recovery status of symptoms and signs, such as pain, percussion pain, loosening, gum swelling, and sinus tract after tooth surgery, was systematically evaluated, and the occurrence of adverse prognosis, such as inflammation recurrence and abnormal occlusal function, was counted to determine the clinical healing effect on the teeth. All clinical outcome assessors, radiographic measurers, and laboratory operators were blinded to group allocation during follow-up and outcome evaluation.

### 1.3 Observation indexes

1.3.1 Clinical efficacy was evaluated according to the guidelines for regenerative endodontic procedures (9). Treatment outcomes were classified as follows: Markedly effective: symptoms of periapical periodontitis had completely resolved, with no gingival inflammation or sinus tract; imaging showed complete healing of the periapical lesion, continued root development, thickening of the root canal walls, and progressive apical closure. Effective: Symptoms of periapical periodontitis were significantly alleviated, and gingival inflammation had largely resolved; imaging showed a marked reduction in the periapical lesion, with evidence of continued root development and narrowing of the root canal. Ineffective: Clinical symptoms showed no improvement or worsened, the periapical lesion showed no improvement or increased in size, and no favorable changes in root development were observed. The overall effectiveness rate was calculated as follows: (markedly effective + effective)/total number of teeth   ×   100%. The clinical efficacy was assessed 6 months after treatment. The classification was based on composite clinical and radiographic criteria. Both resolution or improvement of clinical signs and symptoms and corresponding radiographic improvement were required for classification as “markedly effective” or “effective.” Persistent clinical signs or symptoms or progressive radiographic deterioration resulted in classification as “ineffective,” regardless of improvement in individual outcome parameters.1.3.2 Root growth and development were evaluated using digital measurement of periapical radiographs. Standardized periapical radiographs were obtained using the parallel technique at baseline and 6-month follow-up. A commercial Rinn XCP-DS sensor holder was used to maintain a consistent object–sensor geometry. The exposure parameters were standardized at 60 kVp, 7 mA, and 0.16 s for all patients to minimize inter-visit projection variability. Radiographic measurements were performed using Image-Pro Plus (version 6.0; Media Cybernetics, USA)., Root canal wall thickness was measured at three predefined anatomical levels: (1) the apical third, 2 mm coronal to the anatomical apical foramen; (2) the middle third of the root; and (3) the cervical third of the root. At each level, the mesial and distal canal wall thicknesses were measured and averaged. The mean of the measurements obtained at the three levels was calculated as the overall root canal wall thickness for each tooth. Root length was defined as the straight-line distance from the cementoenamel junction (CEJ) to the anatomical apex.. All radiographic measurements were performed by a calibrated pediatric dentist blinded to the treatment group allocation. Before formal measurements, 20 randomly selected radiographs were measured twice at a 2-week interval to assess intra-examiner reliability, and a second independent examiner measured the same radiographs to assess inter-examiner reliability. The intraclass correlation coefficients (ICCs) were 0.92 for intra-examiner reliability and 0.88 for inter-examiner reliability, indicating excellent measurement reliability.1.3.3 Periodontal indicators: A periodontal index probe was used to detect sulcus bleeding index (SBI) before treatment and 6 months after treatment. The probe tip was inserted into the sulcus by 1–2 mm and slid slowly. Bleeding was observed within 30 s. The highest scores of the buccal and lingual surfaces were recorded and scored according to the 0–4 score standard. The plaque index (PLI) combined with the naked eye or 2% fuchsin basic staining was used to observe the distribution of plaque near the gingival margin. The results of the most plaque on the cheek and tongue were obtained, and the score was 0–3. The periodontal probing depth (PD) was measured by gently probing the periodontal index probe to the bottom of the bag along the long axis of the tooth with a probing force of 20–25 g. Three sites were selected on the buccal and lingual sides, and the mean values were recorded. All periodontal probing measurements were performed by a single calibrated examiner blinded to the group assignment. Interexaminer and intraexaminer reliability were assessed in 20 randomly selected teeth (ICCintra = 0.91, ICCinte*r* = 0.87), showing good reliability.

1.3.4 Gingival crevicular fluid (GCF) collection and cytokine measurement: GCF samples were collected at baseline and 6-month follow-up. Before sampling, supragingival plaque was removed, and the sampling site was isolated using cotton rolls and gently air-dried. Standardized paper-strip sampling was performed by inserting filter paper strips into the gingival crevice of the affected tooth for 30 s. The strips contaminated with blood were discarded. The collected strips were immediately transferred to Eppendorf tubes and stored at −80  °C until analysis. For analysis, the GCF was eluted from the strips by incubation in an assay buffer. The concentrations of basic fibroblast growth factor (bFGF) and vascular endothelial growth factor (VEGF) were measured using commercial enzyme-linked immunosorbent assay (ELISA) kits (Shanghai Enzyme-linked Biological Products; Cat. Nos. ml104876, and ml064255, respectively) according to the manufacturer's instructions. Absorbance was measured at 450 nm using a microplate reader.

1.3.5 Complications including alveolar bone inflammatory absorption, root fracture, and gingival abscess were recorded at the 6-month follow-up. All complication assessments were completed by calibrated examiners blinded to group allocation. Alveolar bone inflammatory absorption was identified and quantified on standardized parallel-technique periapical radiographs, which manifested as progressive reduction of alveolar bone height or expanded periapical radiolucent area compared with baseline images. Root fracture was confirmed by combining clinical examination (abnormal tooth mobility, percussion tenderness) and radiographic findings of visible fracture lines. Gingival abscess was diagnosed based on clinical manifestations including localized gingival swelling, fluctuance, purulent exudate, and corresponding periodontal-periapical imaging features. The number of teeth presenting each type of complication in Group A and Group B was counted respectively. The total complication incidence was calculated as the number of teeth with ≥1 complication divided by the total number of teeth in the group   ×  100%.

1.3.6 Prognosis grouping and analysis of influencing factors according to the final outcome of 6-month follow-up, 148 children were divided into good and poor prognosis groups. In the good-prognosis group, the clinical symptoms disappeared completely, periapical lesions healed, the root continued to develop, and there was no recurrence of inflammation. In the poor-prognosis group, symptoms such as swelling and pain, sinus tract recurrence, apical lesion expansion, root growth length, and root canal wall thickness were significantly slower than those of normal permanent teeth in the same period, and development stagnation occurred, which required secondary intervention treatment. The clinical data of the two groups were collected: gender, age, body mass index, place of residence, course of disease, etiological classification, permanent tooth type, Nolla stage (10), treatment method, apical lesion range, and degree of apical foramen opening. The periapical lesion area was measured on standardized parallel-technique periapical radiographs using Image-Pro Plus 6.0. The lesion boundary was manually traced along the radiopaque-radiolucent transition zone of the periapical bone loss. The software automatically calculated the 2-dimensional lesion area (mm^2^). All tracing operations were completed by the same blinded, calibrated examiner mentioned above. The data of the two groups were compared, and statistically significant indicators were included in the multivariate logistic regression model to screen the prognostic factors.

### 1.4 Statistical methods

S Statistical analyses were performed using SPSS version 26.0. The normality of the continuous variables was assessed using the Shapiro–Wilk test. Normally distributed continuous variables are expressed as mean ± standard deviation and were compared between groups using the independent-samples t-test and within groups using the paired t-test. Non-normally distributed continuous variables were expressed as median (interquartile range) and compared using the Mann–Whitney U test for between-group comparisons and the Wilcoxon signed-rank test for within-group comparisons. Categorical variables are expressed as *n* (%) and were compared using the *χ*^2^ test. Fisher's exact test was used when the expected cell frequency was <5. Multivariable binary logistic regression analysis was performed to identify the factors associated with prognosis. A two-sided *P* < 0.05 was considered statistically significant.

## 2 Results

### 2.1 Clinical efficacy comparison between the two study groups

Clinical outcome analysis indicated that Group A achieved a superior total therapeutic effective rate after treatment compared to Group B, and the inter-group difference was statistically significant (*P* < 0.05). The corresponding data are presented in Table 1.

**Table 1:** Clinical efficacy comparison between the two study groups [*n*(%)].

Group	*n*	Markedly effective	Effective	Invalid	Total effective rate
A Group	74	38 (51.35)	29 (39.19)	7 (9.46)	67 (90.54)
B Group	74	31 (41.89)	24 (32.43)	19 (25.68)	55 (74.32)
*χ* ^2^					6.719
*P*					0.009

### 2.2 Changes in root growth and development status before and after treatment in both groups

Post-treatment morphological observation of tooth roots showed that patients in Group A presented thicker root canal walls and longer root length than those in Group B. Statistical analysis confirmed that the above morphological differences were statistically significant (*P* < 0.05), as summarized in Table 2.

**Table 2:** Changes in root growth and development status before and after treatment in both groups ($\bar{x}\pm s$,mm).

Group	*n*	Root canal wall thickness(mm)		Root length (mm)	
		Before treatment	After treatment	Before treatment	After treatment
A Group	74	2.03 ± 0.25	3.00 ± 0.46*	8.08 ± 0.22	8.76 ± 0.42*
B Group	74	2.04 ± 0.18	2.78 ± 0.35*	8.14 ± 0.27	8.58 ± 0.34*
*t*		0.142	3.247	1.472	2.890
*P*		0.887	0.001	0.143	0.004

*P < 0.05, paired t-test vs pre-treatment within the same group (intra-group change). The t- and P-values listed in table rows represent inter-group comparisons of post-treatment outcomes between Group A and Group B.

### 2.3 Variation of periodontal indices in the two groups pre- and post-treatment

A comprehensive assessment of periodontal status after the intervention suggested that Group A had a lower sulcus bleeding index (SBI) and plaque index (PLI), together with a reduced probing depth (PD) compared with Group B. All detected periodontal parameter differences were statistically significant (*P* < 0.05), as shown in Table 3.

**Table 3:** Variation of periodontal indices in the two groups pre- and post-treatment ($\bar{x}\pm s$).

Group	*n*	SBI (score)		PLI (score)		PD (score)	
		Before treatment	After treatment	Before treatment	After treatment	Before treatment	After treatment
A Group	74	3.43 ± 0.23	1.47 ± 0.30*	3.26 ± 0.44	1.37 ± 0.27*	6.19 ± 0.83	3.23 ± 0.63*
B Group	74	3.38 ± 0.19	1.78 ± 0.33*	3.17 ± 0.43	1.76 ± 0.29*	6.33 ± 0.90	3.99 ± 0.71*
*t*		1.312	5.849	1.196	8.425	1.004	6.983
*P*		0.191	<0.001	0.234	<0.001	0.317	<0.001

*P < 0.05, paired t-test vs pre-treatment within the same group (intra-group change). The t- and P-values listed in table rows represent inter-group comparisons of post-treatment outcomes between Group A and Group B.

### 2.4 Levels of angiogenic factors in gingival crevicular fluid before and after treatment

Biochemical detection of gingival crevicular fluid samples after treatment revealed that the expression levels of bFGF and VEGF were significantly higher in Group A than in Group B. There was a statistically significant difference between the two groups (*P* < 0.05), with detailed data provided in Table 4.

**Table 4:** Levels of angiogenic factors in gingival crevicular fluid before and after treatment ($\bar{x}\pm s$,pg/*μ*l).

Group	*n*	bFGF		VEGF	
		Before treatment	After treatment	Before treatment	After treatment
A Group	74	33.07 ± 4.41	40.66 ± 4.43*	26.04 ± 5.21	41.93 ± 6.44*
B Group	74	32.15 ± 3.93	36.09 ± 4.71*	25.78 ± 4.94	38.18 ± 4.57*
*t*		1.339	5.838	0.317	4.092
*P*		0.183	<0.001	0.752	<0.001

*P < 0.05, paired t-test vs pre-treatment within the same group (intra-group change). The t- and P-values listed in table rows represent inter-group comparisons of post-treatment outcomes between Group A and Group B.

### 2.5 Adverse complication occurrence in the two groups after treatment

Follow-up statistics on adverse reactions and complications demonstrated that Group A had a significantly lower complication incidence than Group B, and the statistical difference between groups was notable (*P* < 0.05). The statistical results are presented in Table 5.

**Table 5:** Adverse complication occurrence in the two groups after treatment [*n*(%)].

Group	*n*	Alveolar bone inflammatory absorption	Root fracture	Gingival abscess	Total incidence
A Group	74	1 (1.35)	1 (1.35)	1 (1.35)	3 (4.05)
B Group	74	4 (5.40)	3 (4.05)	3 (4.05)	10 (13.50)
*χ* ^2^					4.132
*P*					0.042

### 2.6 Analysis of baseline data across different prognosis subgroups

Based on the follow-up findings, patients were classified into poor (*n* = 26) and good prognosis groups (*n* = 122). The general clinical characteristics of the two groups were compared. Compared with patients with a good prognosis, those with a poor prognosis had a lower rate of regenerative endodontic treatment and a greater extent of periapical lesions (*P* < 0.05). The details are presented in Table 6.

**Table 6:** Analysis of baseline data across different prognosis subgroups [($\bar{x}\pm s$),n(%)].

Clinical data	Poor prognosis group(*n* = 26)	Good prognosis group(*n* = 122)	*χ*^2^/*t*	*P*
Gender(male/female)	15/11	68/54	0.033	0.855
Age (years)	11.57 ± 2.62	11.97 ± 2.33	0.777	0.438
Body mass index (kg/m^2^)	16.64 ± 1.31	16.52 ± 1.95	0.299	0.765
Residence			0.064	0.800
Town	14 (53.82)	69 (56.56)		
Countryside	12 (46.15)	53 (43.44)		
Course of disease (d)	6.46 ± 1.20	6.43 ± 1.14	0.255	0.799
Etiological classification			0.030	0.985
Dental caries	11 (42.31)	50 (40.98)		
Malformed central tip fracture	9 (34.61)	42 (34.43)		
Trauma	6 (23.08)	30 (24.59)		
Permanent tooth type			0.268	0.605
Anterior teeth	15 (57.69)	77 (63.11)		
Premolars	11 (42.31)	45 (36.89)		
Nolla staging			0.015	0.993
Phase 7	7 (26.92)	32 (26.23)		
Phase 8	11 (42.31)	51 (41.80)		
Phase 9	8 (30.77)	39 (31.97)		
Surgical treatment			6.719	0.009
Apexification	19 (73.08)	55 (45.08)		
Regenerative endodontic procedure	7 (26.92)	67 (54.92)		
Apical lesion range (mm)	3.11 ± 0.43	2.90 ± 0.39	2.448	0.016
Apical foramen opening			1.310	0.252
<2mm	10 (38.46)	62 (50.82)		
≥2 mm	16 (61.54)	60 (49.18)		

### 2.7 Analysis of adverse prognostic factors of immature permanent teeth with periapical periodontitis

A two-step variable-selection strategy was applied to analyze the prognostic factors. First, variables with *P* < 0.05 in the univariate analysis were selected as the candidate predictors. Second, clinically important potential confounders, including age and Nolla developmental stage, were included in the multivariable binary logistic regression model regardless of their univariate *P*-values to avoid excluding clinically relevant factors solely on the basis of statistical significance. The dependent variable was prognosis, which was coded as poor prognosis = 1 and good prognosis = 0. The independent variables were coded as follows: treatment modality (apexification = 1; regenerative endodontic procedure = 0), apical lesion extent as a continuous variable (mm), age as a continuous variable (years), and Nolla developmental stage as categorical dummy variables. Model fit was assessed using the Hosmer–Lemeshow goodness-of-fit test and Nagelkerke R^2^. A two-sided *P* < 0.05 was considered statistically significant. Multivariate logistic regression analysis showed that the treatment method and range of apical lesions were the influencing factors of poor prognosis of immature permanent teeth with periapical periodontitis (*P* < 0.05), as shown in Table 7.

**Table 7:** Analysis of adverse factors affecting the prognosis of immature permanent teeth with periapical periodontitis.

Factor	*B*	*SE*	*Wald*	*P*	*OR*	95%*CI*
Surgical treatment	1.406	0.500	7.912	0.005	4.081	1.532∼10.873
Apical lesion range	1.605	0.602	7.117	0.008	4.980	1.531∼16.197

Besides the listed significant predictors, age and Nolla developmental stage were forced into this regression model as clinical confounders but showed no statistical significance (*P* > 0.05). Hosmer-Lemeshow test *χ*^2^ = 7.142, *P* = 0.521; Nagelkerke *R*^2^ = 0.247, indicating acceptable model fitness.

## 3 Discussion

Immature permanent teeth are at a critical stage of tooth tissue development. Dental papilla stem cells are in the apical papilla tissue, and many dental papilla and mesenchymal stem cells are distributed. These cells have excellent proliferation, differentiation, and tissue regeneration potential, which provides an important histological basis for the sustainable development of tooth roots in regenerative endodontic treatment (11, 12). Dental regenerative endodontic procedures take tissue regeneration as the core treatment concept, which can re-establish the physiological blood circulation of the root canal system, promote the continuous development of tooth hard tissue, compensate for the deficiency of traditional surgery that cannot repair dental pulp tissue, and ensure the normal development of root morphology and structure of immature permanent teeth from the biological level (13).

The results of this study showed that after treatment, the total effective rate of group A was higher than that of group B, the thickness of the root canal wall was thicker than that of group B, and the length of root was longer than that of group B, suggesting that regenerative endodontic procedure was associated with better short-term treatment outcomes of immature permanent teeth with periapical periodontitis, effectively promoting the growth of the root and the thickening of the root canal wall, and is conducive to the sustainable development of the root. After root canal debridement, regenerative endodontic procedures can induce periapical vascular bleeding, resulting in the formation of blood clots. This structure can be used as a natural biological scaffold to provide a stable microenvironment for the migration, colonization, and differentiation of dental stem cells and mesenchymal stem cells in the apical region and fully retain the tissue regeneration potential of immature permanent teeth (14). Dental pulp blood circulation can deliver nutrition for root development, relieve the inhibition of inflammation on dental papilla stem cells, and ensure the normal root growth. Avoiding excessive mechanical preparation of the root canal during treatment can also protect the root canal wall and periapical tissue structure, creating favorable conditions for root development and lesion repair and improving the treatment effect (15). Caleza-Jiménez (16) reported that six months after a regenerative endodontic procedure, the root of the affected tooth continued to grow, and the thickness of the dentin increased synchronously, suggesting that regenerative endodontic procedures can more effectively promote the development of the root of necrotic immature permanent teeth, which is similar to the results of this study.

Periodontal status is an important indirect index that reflects the control of periapical inflammation. VEGF and bFGF are core cytokines that regulate angiogenesis and tissue repair and play key roles in pulp regeneration and apical bone repair (17). The results showed that SBI and PLI in group A were lower than those in group B, PD was shorter than that in group B, bFGF and VEGF were higher than those in group B, and the incidence of complications in group A was lower than that in group B, suggesting that regenerative endodontic procedures can more effectively control periapical inflammation, improve periodontal health, promote tissue regeneration by upregulating the expression of angiogenesis and tissue repair-related cytokines, and reduce the risk of postoperative complications. Endodontic revascularization can clear the infected lesions in the root canal and reconstruct the physiological blood supply of the root canal, effectively blocking the continuous leakage of inflammatory media in the root canal to the gingival sulcus and periodontal tissue, reducing local inflammatory infiltration and plaque stimulation, and improving periodontal-related indicators, such as gingival sulcus bleeding and periodontal probing depth (18). The blood clot in the root canal induced by this treatment can be used as a biological scaffold to guide periapical stem cells to migrate, proliferate, and differentiate into functional cells, improve local tissue activity, and promote the synthesis and release of VEGF and bFGF. These two types of cytokines can coordinate the regulation of local angiogenesis and the repair process of soft tissue and bone tissue and accelerate bone reconstruction of periapical defects and repair of damaged periodontal tissue (19). Compared with the mode of root canal disinfection and apical mechanical sealing that relies only on drugs, endodontic revascularization can reshape the biological microenvironment inside the root canal, promote thickening of the root canal wall, and enhance the strength of the tooth hard tissue and the defense ability of the periodontal tissue. Simultaneously, complete control of periapical inflammation can reduce the damage of inflammatory media to periodontal tissue, improving the defense ability of periodontal tissue. and reduce the risk of postoperative complications, such as inflammation recurrence, gingival abscess, tooth fracture, and apical absorption (20). Li et al. (21) found that the total effective rate of endodontic revascularization in the treatment of periapical lesions of immature permanent teeth was higher than that of apexification, and the increase inroot length and root area was better, which was similar to the results of this study. However, this study only compared clinical efficacy and did not further explore the relevant risk factors affecting treatment outcomes. Therefore, this study added an analysis of prognostic factors to further enrich the research dimension.

The results of this study showed that the proportion of regenerative endodontic procedures in the poor-prognosis group was less than that in the good-prognosis group, and the range of apical lesions in the poor-prognosis group was greater than that in the good-prognosis group. Further multivariate regression analysis found that the treatment method and range of apical lesions were influencing factors of the poor prognosis of immature permanent teeth with periapical periodontitis. However, caution should be exercised when interpreting these associations. Since treatment allocation was non-randomized in this retrospective cohort, the observed correlation may partially originate from baseline differences among teeth/patients assigned to different treatments rather than representing a pure causal effect of the treatment itself. The risk of poor prognosis in children receiving apexification was 4.081 times higher than that in children receiving regenerative endodontic procedures, and the risk of poor prognosis increased by 4.980 times for every 1 mm increase in the range of apical lesions. This suggests that the choice of surgical treatment and the severity of preoperative apical lesions are the two core factors determining the treatment outcomes. Dental regenerative endodontic procedures rely on the stem cell regeneration mechanism to rebuild the root canal blood supply, which can continuously activate local tissue repair potential, promote periapical bone healing and normal root development, have a good intervention effect on different degrees of inflammation, and effectively reduce the probability of adverse outcomes such as inflammation recurrence and root development stagnation (22). Apexification can only rely on drugs to close the apical foramen, but cannot restore the biological activity of the root canal. The overall repair ability is limited. When the range of apical lesions expands, the local inflammatory load and degree of bone destruction increase. Conventional drug induction makes itdifficult to completely control infections. Inflammation is easy to continue and hinders tissue repair. Combined with the fact that the treatment itself cannot promote root development, the risk of poor prognosis in such children s eventually increases (23).

Within the limitations of this single-center retrospective study with a 6-month follow-up, regenerative endodontic procedures were associated with relatively favorable short-term outcomes in teeth with extensive periapical lesions. However, the present findings alone do not support a definitive recommendation for regenerative endodontic procedures as first-line treatment for cases with large periapical lesions, given the potential for treatment-selection bias and the limited follow-up duration. Therefore, clinical decision-making should integrate the available evidence with individual patient and tooth characteristics.. In clinical practice, thorough intraoperative debridement and standardized blood clot induction should be performed. Postoperatively, oral health education and regular follow-up should be reinforced, and guardians should be instructed to maintain adequate oral hygiene and ensure timely follow-up. Root development and periapical bone healing should be monitored dynamically to facilitate early detection and management of potential complications..This study has several limitations. First, this was a single-center retrospective cohort study with consecutive enrollment of eligible patients between January 2022 and December 2025, and no prespecified prospective sample size calculation was performed. The treatment allocation was non-randomized, and treatment-selection bias could not be fully excluded. Although baseline comparisons indicated that the groups were generally comparable with respect to major clinical characteristics, residual and unmeasured confounding factors may have influenced the observed associations. Second, the follow-up period was limited to 6 months; therefore, the findings reflect only the short-term outcomes. Longer-term outcomes, including continued root maturation, tooth fracture resistance, recurrence of periapical inflammation, and biological responses of the pulp–dentin complex could not be evaluated. Third, the root canal irrigation protocol used 3% hydrogen peroxide combined with normal saline rather than the sodium hypochlorite-based protocol recommended in the AAE regenerative endodontic guidance, which may have influenced the infection control and stem cell viability. Fourth, the multivariable logistic regression model included variables identified through univariate analysis, together with selected clinically relevant covariates, including age and Nolla developmental stage. Nevertheless, other unmeasured clinical or biological confounders may have affected the prognosis. Fifth, radiographic assessments were based on two-dimensional periapical radiographs rather than three-dimensional CBCT-based volumetric measurements, which may have introduced a measurement bias, particularly in the assessment of periapical lesions. Future multicenter, large-sample prospective studies with longer follow-up periods and standardized disinfection protocols are required to validate these findings.

In conclusion, regenerative endodontic procedure was associated with favourable short-term (6-month) outcomes for immature permanent teeth with periapical periodontitis, promoting short-term root development and repair, improving the periodontal microenvironment andlevel of related repair factors, and reducing the incidence of complications. Surgical treatment and the range of apical lesions were the key factors affecting the prognosis of patients. Surgical treatment can be selected according to the situation of apical lesions, and individualized treatment can be implemented to improve the prognosis of children.

This retrospective observational study was reported following the STROBE (Strengthening the Reporting of Observational Studies in Epidemiology) checklist for cohort studies.

## Data Availability

The original contributions presented in the study are included in the article/Supplementary Material, further inquiries can be directed to the corresponding author.
